# Using Chaos-Game-Representation for Analysing the SARS-CoV-2 Lineages, Newly Emerging Strains and Recombinants

**DOI:** 10.2174/0113892029264990231013112156

**Published:** 2023-11-22

**Authors:** Amarinder Singh Thind, Somdatta Sinha

**Affiliations:** 1 Department of Biological Sciences, Indian Institute of Science Education & Research, Mohali, India;; 2 Illawarra Shoalhaven Local Health District (ISLHD), NSW Health, Australia

**Keywords:** CGR, alignment-free method, SARS-CoV-2, lineages A and B, XBB strain, phylogeny, CGRphylo, maximum-likelihood

## Abstract

**Background:**

Viruses have high mutation rates, facilitating rapid evolution and the emergence of new species, subspecies, strains and recombinant forms. Accurate classification of these forms is crucial for understanding viral evolution and developing therapeutic applications. Phylogenetic classification is typically performed by analyzing molecular differences at the genomic and sub-genomic levels. This involves aligning homologous proteins or genes. However, there is growing interest in developing alignment-free methods for whole-genome comparisons that are computationally efficient.

**Methods:**

Here we elaborate on the Chaos Game Representation (CGR) method, based on concepts of statistical physics and free of sequence alignment assumptions. We adopt the CGR method for classification of the closely related clades/lineages A and B of the SARS-Corona virus 2019 (SARS-CoV-2), which is one of the fastest evolving viruses.

**Results:**

Our study shows that the CGR approach can easily yield the SARS-CoV-2 phylogeny from the available whole genomes of lineage A and lineage B sequences. It also shows an accurate classification of eight different strains and the newly evolved XBB variant from its parental strains. Compared to alignment-based methods (Neighbour-Joining and Maximum Likelihood), the CGR method requires low computational resources, is fast and accurate for long sequences, and, being a K-mer based approach, allows simultaneous comparison of a large number of closely-related sequences of different sizes. Further, we developed an R pipeline CGRphylo, available on GitHub, which integrates the CGR module with various other R packages to create phylogenetic trees and visualize them.

**Conclusion:**

Our findings demonstrate the efficacy of the CGR method for accurate classification and tracking of rapidly evolving viruses, offering valuable insights into the evolution and emergence of new SARS-CoV-2 strains and recombinants.

## 1 INTRODUCTION

Viruses are one of the fastest evolving organisms, having up to a million times higher mutation rates than their host in some cases [1]. They are known to cause outbreaks of multiple diseases in both prokaryotes and eukaryotes [2]. The recent pandemic of COVID-19 [3] and the Ebola outbreak reported in 2022 in the Western Region and Central Region of Uganda [4] are the two latest examples of their tremendous health and socioeconomic hazards. Viruses are easily transmitted between hosts, and the rapid evolution rates of viral genomes help in their fast adaptation and survival in diverse and challenging environments.

SARS-CoV-2 is among the fastest-evolving viruses [5]. The SARS-CoV-2 was detected in Wuhan, China [6], and transmitted very fast across the world, causing the coronavirus disease 2019 (COVID-19) pandemic in humans. Around a 1 - 4% death rate is reported on closed cases on worldwide statistics, which varies with age [7]. Various researchers have studied its evolution across the globe, but tracking its lineage has been a challenging task because of the rapid mutation of its genome. It is complicated to track the source, largely due to asymptomatic patients. Studying the virus phylogeny based on its whole genome sequence characteristics in both symptomatic and asymptomatic hosts can help track more accurate evolving patterns.

Furthermore, recombinant forms of SARS-CoV-2 exist, which carry genomic regions from different parental lineages. The Pango Network (https://www.pango.network/), which is an open collaboration of scientists around the world, has catalogued 49 recombinant variants of SARS-CoV-2. Accurate classification of the recombinant forms is essential for understanding the virus evolution under specific selection pressures and is also important for the design of therapeutic applications (ICTVE Viruses Committee) [8, 9]. Viruses with high mutation rates tend to carry drug-resistant mutations, and the treatment of infected cases often require multiple drug therapy. Timely and accurate evolutionary classification and tracking of the recombinant forms can provide us with more insights into the evolution of recombinant infection and the corresponding medications. One of the more prominent of the newer and emerging recombinant lineages is XBB, which combines BJ.1 and BA.2.75. This lineage and its sub-lineage XBB.1 are studied to be the most antibody-evasive variants, which are capable of evading a wide variety of monoclonal antibodies and antibodies raised by vaccination, infection, and combinations [10]. Due to XBB's ability to evade a wide range of neutralizing antibodies, it appears to have an advantage over the earlier variants.

However, the evolutionary classification of viruses, which suggests common ancestors, is not easy, and the classification system has changed from time to time. The most used Baltimore classification system is based on the nucleic acid type and replication mode [11]. With the advancement of technology in sequence analysis, molecular phylogeny is widely used to construct evolutionary phylogenetic trees. Molecular phylogeny is based on the analysis of molecular differences at the genomic and sub-genomic levels to infer evolutionary histories and relationships among the sequences [12]. Mainly, multiple sequence alignment approaches (*e.g*., MUSCLE [13], MAFFT [14], Clustal Omega [15] and ClustalW [16]) are used, which align sets of homologous proteins or genes. Different clustering approaches (*e.g*., neighbor-joining, maximum likelihood, maximum parsimony, and Bayesian analysis) measure the similarity between the aligned sequences to construct a phylogenetic tree.

However, alignment-based approaches make certain assumptions, which are not always applicable in the real world [16]. For example, viral genomes are not linearly defined because they evolve rapidly [17] by processes such as horizontal gene transfer, recombinant genetic events, mutations, and gene loss and gain. Thus, the assumption of a linear arrangement of homologous genes for assessing similarity between genomes may be questioned. Moreover, alignment-based approaches usually require *a priori* knowledge of homologous genes in different species to classify them [18, 19]. Therefore, these methods do not address the problems that arise owing to the assumption of the linear arrangement of homologous sequences and dependence on pre-acquired knowledge of the evolution of sequences (*i.e*., conservation of proteins or genes). Furthermore, owing to their computational extensiveness, these approaches are usually not applicable for comparisons at the whole-genome scale [20]. Also, in the case of DNA/RNA, random sequences can show a 50% similarity. Therefore, it is more challenging to address similarity-based issues about DNA/RNA sequences using alignment-based approaches [21, 22].

With advanced and less expensive sequencing technology, a large quantity of whole-genome sequence data is available at present and growing at an exponential rate. In the recent era, researchers are focusing on the development of alignment-free methods, with a low computational requirement, for the comparison of genetic differences at the whole-genome level. One such alignment-free method is the Chaos Game Representation (CGR), which was initially used for the visualization of DNA sequence patterns in CGR plots [23]. Subsequently, these observed patterns in different genomes were explained in terms of dinucleotide, trinucleotide, and higher nucleotide frequencies [24, 25]. Furthermore, studies have described the use of nucleotide frequencies to determine distances between CGR plots and for phylogenetic analysis [26]. Chaos game representation has also been successfully used for the classification of HIV-1 subtypes [27]. The studies have demonstrated the capability of CGR to capture and correctly classify the intra-species variability in the subtypes and quasi-species. A few other studies also used a combined approach of alignment-free methods and supervised learning for subtyping HIV-1 [28]. In this paper, we use the CGR method for the classification of SARS-CoV-2 lineages, strains and recombinants. Our study shows the CGR-based SARS-CoV-2 phylogeny from the whole genome sequences, the clustering patterns for lineage A and lineage B, and 8 different strains and recombinant XBB among SARS-CoV-2 sequences submitted to the GISAID database. We have also developed a simple, easy to use, open-source package “CGRphylo” in R [29] for these kinds of analysis that can also be used in less-resource educational environments.

## 2 MATERIALS AND METHODS

### 2.1 Datasets

For SARS-CoV-2, we prepared 3 different datasets of whole genome sequences, which were selected randomly from the GISAID database [30, 31]. These sequences of the SARS-CoV-2 were obtained with the filters for human host and complete/high coverage sequences.

The selection of the first dataset was based on the lineages of A and B of the SARS-CoV-2 and the MERS (OL622035.1_MERS-CoV_Riyadh_2016) whole genome sequences as a root. There were 106 sequences in total (47 A, 58 B and a root). The length of the sequences for lineage A ranges from 29653-29900, with a median of 29824. The lineage B sequences length has a standard deviation of 282.1384. The length of the overall sequence ranges from 29653-29994 with median value of 29819 and standard deviation of 63.54, as shown in Fig. (**1**). All the SARS-CoV-2 sequences used in dataset 1 are reported in Supplementary Table **1** with corresponding GISAID accession number.

The second dataset merges 69 random sequences of 8 strains with metadata information obtained from GISAID. These sequences were obtained after filtering the initial dataset with ‘n/N’ (unknown nucleotide residue) nucleotide bases > 50. That means, any sequence that contained a considerable number of 'n/N' bases (exceeding a threshold of 50) was removed from the dataset. These 8 strains include Alpha, Beta, Gamma, Delta, Omicron, Lambda, GH/490R (490R) and Mu. The characteristics of these sequences are shown in Fig. (**1**). All the SARS-CoV-2 sequences used in dataset 2 are reported in Supplementary Table **2**.

The third dataset is composed of a total of 50 sequences of Omicron sub-lineages, BJ.1 and BA.2.75, and the recently discovered XBB, a recombinant lineage between the two Omicron sub-lineages, BJ.1 and BA.2.75. All the SARS-CoV-2 sequences used in dataset 3 are reported in the Supplementary Table **3**. Copy of FASTA files used in this study is also available on CGRphylo GitHub page https://github.com/amarinderthind/CGRphylo.

### 2.2 Chaos Game Representation (CGR) Algorithm

A CGR plot [23] of any genomic sequence can be drawn in the form of a square with each of the four nucleotides A, C, G and T occupying one of the four smaller squares as shown in Fig. (**2A**). The plot of any sequence starts at the center of the square. The first nucleotide of the DNA sequence is plotted at the mid-point of the line connecting the center point and the corner corresponding to the first nucleotide of the sequence.

The successive nucleotides of the sequence are plotted as points corresponding to the mid-point between the previous point and the corner vertex of the next nucleotide of the sequence. This process is repeated for the entire DNA sequence for each of the genomes. The CGR plot converts a linear nucleotide sequence to a dot plot, requiring much less space and is useful for the simultaneous comparison of many genomes.

#### 2.2.1 Frequency Matrices Computation and Distance Matrix Calculations

**Fig. (2):**
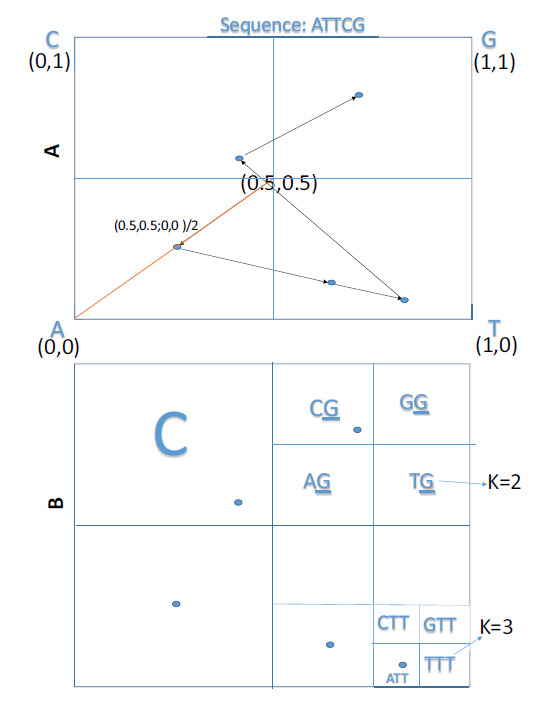
(**A**) Schema for plotting the DNA sequence (ATTCG) in CGR. (**B**) Division of CGR to obtain dimer (K=2), trimer (K=3) and higher nucleotide frequencies.

The method of CGR generates iterative fractals in polygons [32]; hence, in a square, each sub-divided square box is a self-replicating image and thus can be resolved to dimers, trimers, and longer nucleotides sequences (K-mers) for any genome sequence as shown in Fig. (**2B**). The frequency of each K-mer is calculated by dividing the CGR square into a 4^K grid, and the number of points plotted in a particular grid is called the frequency of that K-mer. For example, the frequency of monomer T is 2 (with A, C and G being one) as can be seen from (Figs. **2A** and **B**). The frequencies of dimer CG and trimer ATT are 1 (the grids of the other di- and tri-mers are not shown in the CGR).

Once these frequency matrices are generated for each input sequence, pair-wise distances between various sequences can be computed using any distance methods. A distance matrix between sequences is useful for phylogenetic analysis. At present, in the CGRphylo, three different distance measures have been implemented - the simple *Euclidian* distance, *Euclidean squared* distance, and the *Manhattan* distance. Distance Matrix between two genome sequences is computed by calculating the difference in the frequencies of K-mers from their CGR plots. The results shown in this study are based on Euclidian distances.

Genomic location, where software is not able to make a base, is usually listed as “N” base and significant differences in the whole genome sequence length and the presence of n/N nucleotide bases contributes to frequency bias. Since we are analyzing closely related genomes, to minimize such bias, the end bases are trimmed to obtain the same length of every sequence equal to the minimum length of the sequence in the input dataset. In addition, we filtered SARS-CoV-2 sequences with N nucleotide bases > 50. For further details, refer to Supplementary Document (Supplementary Material for CGR Method).

### 2.3 CGRphylo

CGRphylo (https://github.com/amarinderthind/CGRphylo), the R pipeline developed by us, provides the options to filter/clean the input sequences in the FASTA format and performs initial FASTA file checks using the R package “seqinr” (https://seqinr.r-forge.r-project.org/). Further it generates the CGR plots for each DNA sequence (with a choice for trimming). It performs the K-mer frequency calculations, and then the distance calculations can be performed using any of the distance methods (see section 2.2). For visualization, CGRphylo also implements conversion of the distance matrix into the MEGA/Phylip distances, which are widely used tools in the phylogenetic analysis [33, 34]. The distance matrix can also be visualized with R using integrated packages such as “ape” [35] or “treeio” [36]. The entire pipeline and input data used in this study are available on the GitHub page mentioned above.

## 3 RESULTS AND DISCUSSION

The DNA sequences from the three datasets as per Fig. (**1**) were analyzed using CGRPhylo and cladograms were created. The tool processes multiple FASTA files as per given word-length ‘K’, and pair-wise distance matrix between the sets of input genomes are generated as output. Cladograms are generated using the Neighbor-Joining (NJ) algorithm and are edited, rooted and labelled using the MEGA software.

**Fig. (3):**
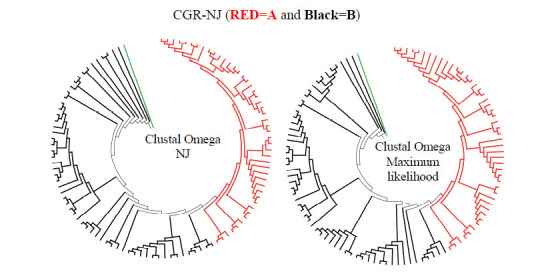
Multiple sequence alignment (MSA) based cladograms of lineage A (in Red) and lineage B (in Black) of SARS Cov-2 using NJ clustering and maximum likelihood method. Green color refers to MERS outlier.

To get an idea of the efficacy of the CGR approach of classification, compared to the commonly used alignment-based grouping of sequences, we first created the cladograms from Dataset 1 sequences – SARS-CoV-2 genome sequences of Lineage A and B. These were aligned using Multiple Sequence Alignment (MSA) methods in Clustal Omega [37], and trees were obtained using NJ and the Maximum Likelihood approaches (Fig. **3**). It may be noted that, even though Lineage A and B sequences cluster separately, they do not fall into two clearly resolved major subgroups (annotation based on GISAID) in this method.

### 3.1 Word Length Estimation

Earlier studies on CGR classification of HIV (Human Immunodeficiency Virus) whole genomes have shown that a minimum word-length of K = 6 was necessary to classify the different HIV subtypes and the sub-subtypes [27). A similar approach is used for the SARS-CoV-2 Lineage A and B genome sequences. Fig. (**4**) show the cladograms for K = 3, 4, 5, and 6. The upper row of Fig. (**4**) shows the cladograms of SARS-CoV-2 Lineage A and B sequences classified at lower word-lengths *i.e*., K = 3 and 4. It is clear that these word lengths were unable to separate Lineage A and B into two separate major groups.

Clear group separation was detected at higher word-lengths, for K ≥ 5. Fig. (**4**) (lower row) shows the cladograms where this method successfully separates and clearly resolves the two major subgroups, Lineage A from Lineage B, for K = 5 and K = 6. So, K = 5 or 6 is established as the standard word-length for this analysis, which successfully separates the two major lineages A and B. On comparing Figs. (**3** and **4**), it is clear that the CGR approach resolves the SARS Cov-2 lineages more accurately than the usual alignment-based clustering approaches.

### 3.2 Classification of SARS-CoV-2 Strains

**Fig. (5):**
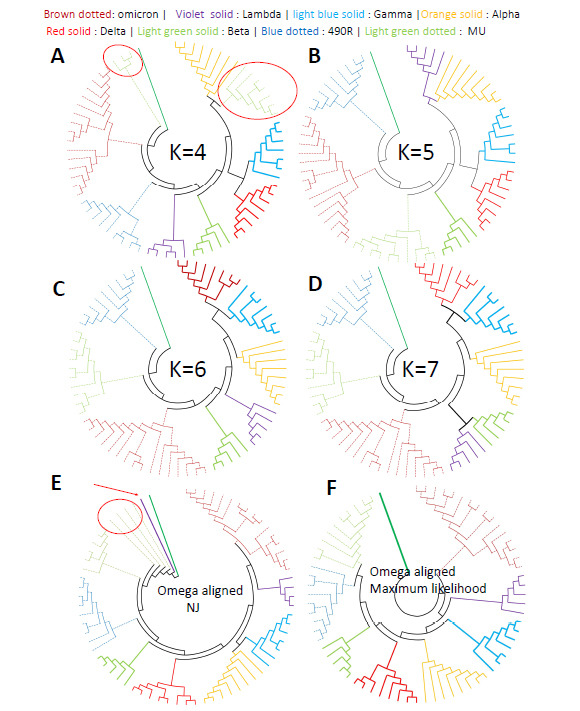
Cladogram of 8 different strains of SARS-CoV-2 using - (**A**) K=4, (**B**) K=5, (**C**) K=6, (**D**) K=7, (**E**) Multiple sequence alignment (MSA) and NJ clustering, and (**F**) MSA-Maximum likelihood method.

For Dataset 2, eight strains of the SARS-CoV-2 were first classified at a lower word length (K = 4). Fig. (**5A**) shows that 7 out of 8 strains are accurately classified into separate groups, except the sequences of the MU strain (circled). For higher word lengths (K = 5 and more), the cladograms show clear separation of the sequences belonging to different strains (Figs. **5B**-**D**). In the multiple sequence alignment-based MSA-NJ approach, however, one sequence (Lambda_C.37_O_Illumina_Chile345) is found to cluster incorrectly (arrow in Fig. **5E**). Among the Lambda strain sequences, this sequence is an outlier in the Box plot in Fig. (**1**). It differs from others as it belongs to a different clade 'O', while the others belong to the Lambda 'GR' type. However, the MSA-Maximum Likelihood approach classified all strain types into 8 separate groups (Fig. **5F**).

### 3.3 Classification of SARS-CoV-2 Recombinant Strain

**Fig. (6):**
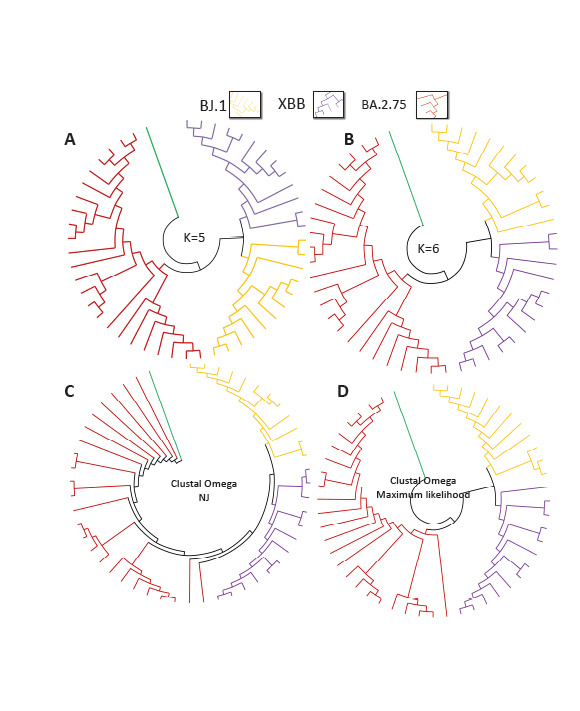
Cladogram of the recombinant XBB of Omicron sub lineages (BJ.1 and BA.2.7.5) and its parental sequences (**A**) K=5, (**B**) K=6. Cladograms from (**C**) MSA-NJ, and (**D**) MSA-Maximum likelihood method.

Like the earlier datasets, Dataset 3 of the Omicron sub-lineages and their recombinant XBB were first classified at lower word lengths (*i.e*., K = 4). Interestingly, K = 4 did classify them into three separate major groups. However, lower word-length, K = 3, misclassified 2 sequences of the XBB (figure not shown). For higher word lengths, correct cladograms are formed as expected (Figs. **6A** and **B**). The cladograms from the MSA based approaches showed correct groupings of the 3 major groups by the Maximum Likelihood approach (Fig. **6D**), but the NJ clustering separated 3 sub lineages and the recombinant, but the BA.2.7.5 sub-lineages sequences are scattered (Fig. **6C**).

### 3.4 Comparison of CGRphylo and Clustal-omega Computation Times

We conducted a comparison of computational times between CGRphylo and Clustal-Omega (1.2.4 ubuntu-x86_64) command line tool for multiple sequence alignments (MSA). Clustal-Omega is one of the fastest MSA tools. The results are shown in Table **1**. For CGRphylo, the most computationally intensive step is the frequency calculation, whereas for alignment-based methods, such as, Maximum Likelihood and NJ, it is the MSA that is time intensive and consumes the most time. To ensure a fair comparison, we utilized the same Linux Machine with a configuration of 32 GB RAM and an i7-10700 CPU @ 2.9 GHz x 16, and set similar computing parameters, such as using 1 CPU. The results in Table **1** demonstrate that CGRphylo efficiently processed 69 SARS-CoV-2 genomes (Dataset 2) 5 times faster than the time taken by Clustal-Omega to complete the same task. For Dataset 1 (106 genomes), this difference grew to 13.7 times (Table **1**). The significant difference in processing time highlights the advantage of CGRphylo for rapid and efficient analysis of large datasets.

In the context of MSA, the computational cost increases as additional sequences require pairwise comparisons with all other sequences, making the process more computationally intensive as the dataset grows. However, this is not the case for CGRphylo. Addition of one sequence simply requires the addition of one dot-plot (CGR) and its frequency and distance calculations are not computationally expensive.

Based on our experience and analyses, we recommend using k = 5 or 6 for SARS-CoV-2 analyses, as these K-mer sizes, or even higher, have shown the ability to accurately resolve phylogeny.

## CONCLUSION

Our results demonstrate that CGR can accurately classify closely related viral genomes, such as the SARS-CoV-2 A and B lineages, its newly emerging strains and their recombinants. Three datasets were analyzed, two of which were for SARS-Cov-2. In the SARS Cov-2 analysis using CGR methods, the method successfully clustered lineages A and B into separate groups. In addition, we tested this method for the separation of eight different strains of the SARS-CoV-2 and found that it was capable of doing so accurately at k ≥5 in a shorter timeframe as compared to methods based on Multiple Sequence Alignment.

Further, CGRphylo could classify XBB recombinant of SARS-CoV-2 Omicron lineage easily, and all known sequences of the recombinants clustered properly for length K = 5 and 6. It was observed that the MSA-NJ approach was able to segregate the sequences, but it did not effectively group them into the expected major groups (BJ.1, BA.2.7.5, and XBB). Specifically, the BA.2.7.5 sequences were not clustered as accurately as the other two groups. Instead of being part of one major group, multiple groups of BA.2.7.5 were observed. However, 3 large clusters were formed by the MSA-Maximum Likelihood approach.

The CGRphylo works accurately for SARS-CoV-2 lineages, SARS-CoV-2 strains, and also on the recombinants of the sub lineages. Hence, we can track new emerging SARS-CoV-2 strains or recombinants with larger genome length in any newly emerging or unknown SARS-CoV-2 sequences. It can analyse big datasets with much less computational power compared to any alignment-based approach. The CGRphylo will also be useful in academically low resource settings such as classrooms and field stations.

## Figures and Tables

**Fig. (1):**
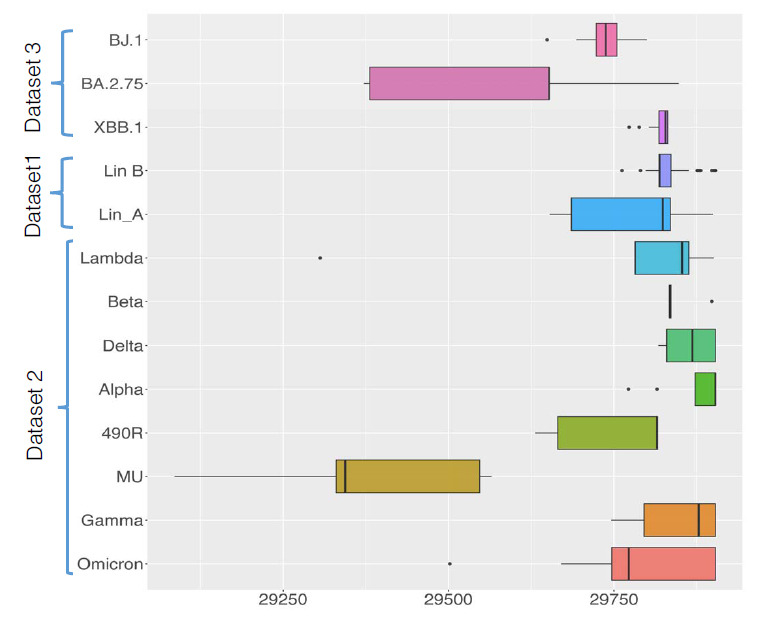
The Box-Whisker plots show the median ranges for the three datasets. Dataset 1 is lineage-based and has sequences of Lineage A and Lineage B. Dataset 2 contains the sequences of 8 different SARS-CoV-2 variants. Dataset 3 is composed of two sub-lineages of Omicron and its recombinant XBB. The length of the genome/s (represented in the X-axis) shows that the MU variant genome is shorter than other types. Omicron and Lambda each have one outlier. Where dots are data points and the solid line within the box is the Median.

**Fig. (4):**
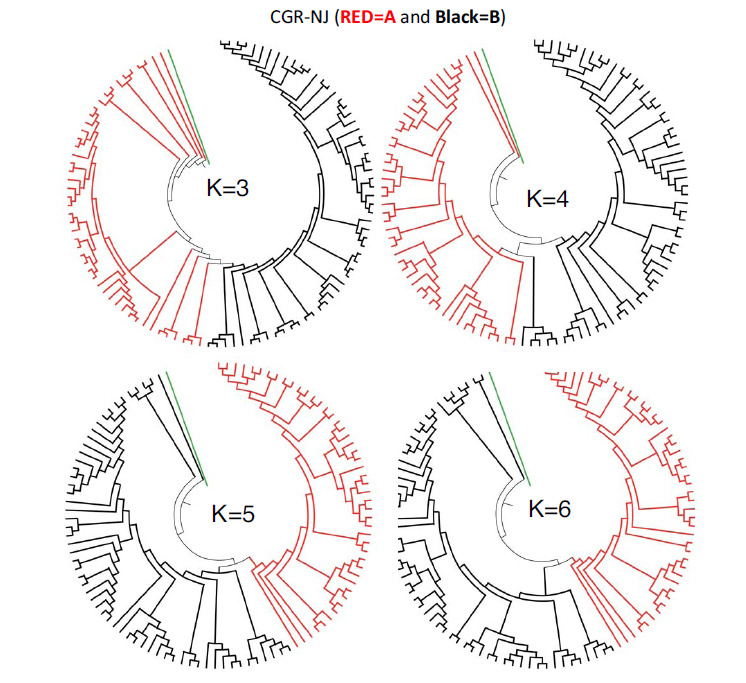
Cladogram of lineage A (in Red) and lineage B (in Black) of SARS Cov-2 using K = 3, K = 4, K = 5 and K = 6. Green color refers to MERS outlier.

**Table 1:** Computational time comparison of CGRphylo and clustal-omega.

**Datasets (Number of Genomes)**	**CGRphylo (K=5) Processing Time**	**Clustal-omega Processing Time**
Datasets 2 (69 Genomes)	4 minute 48 sec	57 minute
Dataset 1 (106 Genomes)	7 minute 18 sec	1 hour, 40 minute

## Data Availability

The CGRphylo pipeline and datasets analysed for this study are available at https://github.com/amarinderthind/CGRPhylo

## LIST OF ABBREVIATIONS

CGR: Chaos Game Representation
COVID-19: Coronavirus Disease 2019
HIV: Human Immunodeficiency Virus
MSA: Multiple Sequence Alignment
NJ: Neighbor-Joining
