# Supplementary material for: Using Chaos-Game-Representation for Analysing the SARS-CoV-2 Lineages, Newly Emerging Strains and Recombinants
Source: Curr Genomics. 2023 Nov 22;24(3):187–95. doi: 10.2174/0113892029264990231013112156 (PMC10761335; doi:10.2174/0113892029264990231013112156)
Supplement: Supplementary file 1 [file CG-24-187_SD1.pdf]

Supplementary Material

Using Chaos-Game-Representation for Analysing the SARS-CoV-2 Lineages, Newly Emerging Strains and Recombinants

Amarinder Singh Thind[1,2] and Somdatta Sinha[1,*]

[1]Department of Biological Sciences, Indian Institute of Science Education & Research, Mohali, India; [2]Illawarra Shoalhaven Local Health District (ISLHD), NSW Health, Australia

SUPPLEMENTAL TABLE

Data Availability

GISAID Identifier: EPI_SET_230531ds
doi: 10.55876/gis8.230531ds

All genome sequences and associated metadata in this dataset are published in GISAID's EpiCoV database. To view the contributors of each individual sequence with details such as accession number, Virus name, Collection date, Originating Lab and Submitting Lab and the list of Authors, visit 10.55876/gis8.230531ds

Data Snapshot

- EPI_SET_230531ds is composed of 128 individual genome sequences.
- The collection dates range from 2020-01-31 to 2022-09-28;
- Data were collected in 3 countries and territories;
- All sequences in this dataset are compared relative to hCoV-19/Wuhan/WIV04/2019 (WIV04), the official reference sequence employed by GISAID (EPI_ISL_402124). Learn more at https://gisaid.org/WIV04.

Supplementary Table 1. Dataset 1 composed of 106 SARS-Cov-2 random sequences of Lineage A and Lineage B obtained from GISAID (Name annotated with GISAID metadata information).

GISAID	length	n_base	a_base	g_base	t_base	c_base	GC_content	Lineage	Names
EPI_ISL_413523	29851	0	8915	5862	9589	5485	38.0121269	A	Lin-A-In-MH-1-31-2020-EPI_ISL_413523-2020-01-31
EPI_ISL_435111	29866	8	8917	5859	9595	5486	37.98633898	A	Lin-A-In-DL-NCDC-02415-2020-EPI_ISL_435111-2020-03-30
EPI_ISL_450324	29829	0	8907	5852	9586	5484	38.00328539	A	Lin-A-In-MH-CCMB-NIV4-2020-EPI_ISL_450324-2020-03-26
EPI_ISL_452202	29805	0	8902	5852	9574	5477	38.01040094	A	Lin-A-In-MH-NIV-2810-2020-EPI_ISL_452202-2020-03-16
EPI_ISL_452203	29805	0	8904	5848	9575	5478	38.00033551	A	Lin-A-In-MH-NIV-4367-2020-EPI_ISL_452203-2020-03-22
EPI_ISL_452204	29799	0	8904	5847	9573	5475	37.99456358	A	Lin-A-In-MH-NIV-5109-2020-EPI_ISL_452204-2020-03-26
EPI_ISL_452205	29805	0	8904	5847	9576	5478	37.99698037	A	Lin-A-In-MH-NIV-5119-2020-EPI_ISL_452205-2020-03-26
EPI_ISL_455016	29836	0	8912	5852	9586	5486	38.00107253	A	Lin-A-In-GJ-GBRC119-2020-EPI_ISL_455016-2020-04-27
EPI_ISL_455645	29858	0	8919	5858	9590	5491	38.00991359	A	Lin-A-In-WB-S15-2020-EPI_ISL_455645-2020-04-03
EPI_ISL_455649	29848	0	8913	5858	9590	5487	38.00924685	A	Lin-A-In-WB-S19-2020-EPI_ISL_455649-2020-04-20
EPI_ISL_455658	29873	0	8928	5857	9598	5490	37.98413283	A	Lin-A-In-WB-S29-2020-EPI_ISL_455658-2020-04-30
EPI_ISL_455671	29858	0	8919	5857	9591	5491	38.0065644	A	Lin-A-In-WB-S51-2020-EPI_ISL_455671-2020-05-03
EPI_ISL_455761	29660	0	8857	5810	9538	5455	37.98044504	A	Lin-A-In-OR-RMRC160-2020-EPI_ISL_455761-2020-05-07
EPI_ISL_455763	29843	0	8914	5853	9588	5488	38.00221157	A	Lin-A-In-OR-RMRC163-2020-EPI_ISL_455763-2020-05-07
EPI_ISL_455764	29686	0	8865	5815	9551	5455	37.96402345	A	Lin-A-In-OR-RMRC164-2020-EPI_ISL_455764-2020-05-07
EPI_ISL_455766	29685	0	8865	5814	9551	5455	37.96193364	A	Lin-A-In-OR-RMRC166-2020-EPI_ISL_455766-2020-05-07
EPI_ISL_455767	29664	0	8857	5812	9546	5449	37.96183927	A	Lin-A-In-OR-RMRC167-2020-EPI_ISL_455767-2020-05-07
EPI_ISL_455770	29655	0	8854	5809	9544	5448	37.95987186	A	Lin-A-In-OR-RMRC171-2020-EPI_ISL_455770-2020-05-07
EPI_ISL_461482	29855	0	8921	5859	9589	5486	38.00033495	A	Lin-A-In-GJ-GBRC153b-2020-EPI_ISL_461482-2020-04-27
EPI_ISL_463024	29673	0	8860	5815	9547	5451	37.96717555	A	Lin-A-In-OR-ILSCV13907-2020-EPI_ISL_463024-2020-05-10
EPI_ISL_463026	29784	0	8895	5846	9566	5477	38.01705614	A	Lin-A-In-OR-ILSCV13929-2020-EPI_ISL_463026-2020-05-10
EPI_ISL_463029	29658	0	8856	5810	9540	5452	37.97289096	A	Lin-A-In-OR-ILSCV14262-2020-EPI_ISL_463029-2020-05-11
EPI_ISL_463033	29653	0	8853	5808	9540	5452	37.97254915	A	Lin-A-In-OR-ILSCV14831-2020-EPI_ISL_463033-2020-05-10
EPI_ISL_463034	29658	0	8855	5810	9542	5451	37.96951919	A	Lin-A-In-OR-ILSCV14847-2020-EPI_ISL_463034-2020-05-10
EPI_ISL_463058	29775	0	8892	5843	9562	5478	38.02183039	A	Lin-A-In-OR-ILSCV17741-2020-EPI_ISL_463058-2020-05-13
EPI_ISL_463064	29655	0	8855	5809	9539	5452	37.97336031	A	Lin-A-In-OR-ILSCV19048-2020-EPI_ISL_463064-2020-05-16
EPI_ISL_463073	29658	0	8856	5811	9540	5451	37.97289096	A	Lin-A-In-OR-ILSCV20165-2020-EPI_ISL_463073-2020-05-05
EPI_ISL_463074	29672	0	8860	5814	9542	5456	37.98193583	A	Lin-A-In-OR-ILSCV20166-2020-EPI_ISL_463074-2020-05-05
EPI_ISL_469043	29815	0	8906	5848	9579	5482	38.0010062	A	Lin-A-In-GJ-GBRC198-2020-EPI_ISL_469043-2020-06-08
EPI_ISL_475035	29829	0	8908	5854	9589	5478	37.98987562	A	Lin-A-In-GJ-GBRC208a-2020-EPI_ISL_475035-2020-06-11
EPI_ISL_481111	29834	0	8909	5855	9585	5485	38.01032379	A	Lin-A-In-OR-ILSCV25378-2020-EPI_ISL_481111-2020-05-19
EPI_ISL_481125	29810	0	8903	5849	9576	5482	38.01073465	A	Lin-A-In-OR-ILSCV28062-2020-EPI_ISL_481125-2020-05-21
EPI_ISL_481131	29658	0	8855	5812	9539	5452	37.9796345	A	Lin-A-In-OR-ILSCV29958-2020-EPI_ISL_481131-2020-05-23
EPI_ISL_481136	29832	0	8908	5854	9585	5485	38.00951998	A	Lin-A-In-OR-ILSCV29971-2020-EPI_ISL_481136-2020-05-23
EPI_ISL_481150	29806	0	8902	5849	9576	5479	38.00577065	A	Lin-A-In-OR-ILSCV31684-2020-EPI_ISL_481150-2020-05-26
EPI_ISL_481151	29853	0	8915	5858	9590	5490	38.01293002	A	Lin-A-In-OR-ILSCV31693-2020-EPI_ISL_481151-2020-05-26
EPI_ISL_481152	29834	0	8908	5856	9586	5484	38.01032379	A	Lin-A-In-OR-ILSCV31694-2020-EPI_ISL_481152-2020-05-26
EPI_ISL_483850	29829	0	8911	5852	9584	5482	37.99658051	A	Lin-A-In-GJ-GBRC259-2020-EPI_ISL_483850-2020-06-11
EPI_ISL_483870	29829	0	8910	5852	9584	5483	37.99993295	A	Lin-A-In-GJ-GBRC272a-2020-EPI_ISL_483870-2020-06-13
EPI_ISL_483872	29829	0	8911	5850	9583	5485	37.99993295	A	Lin-A-In-GJ-GBRC273a-2020-EPI_ISL_483872-2020-06-13

EPI_ISL_483873	29829	0	8907	5855	9585	5482	38.00663784	A	Lin-A-In-GJ-GBRC273b-2020-EPI_ISL_483873-2020-06-13
EPI_ISL_495077	29824	0	8908	5852	9582	5482	38.00295064	A	Lin-A-In-GJ-GBRC319-2020-EPI_ISL_495077-2020-06-17
EPI_ISL_508181	29900	0	8953	5859	9594	5494	37.96989967	A	Lin-A-In-UT-AR33-2020-EPI_ISL_508181-2020-06-02
EPI_ISL_508434	29884	0	8952	5857	9588	5487	37.96011243	A	Lin-A-In-OR-MW30-2020-EPI_ISL_508434-2020-05-26
EPI_ISL_512070	29834	0	8911	5852	9585	5486	38.00362003	A	Lin-A-In-GJ-GBRC334a-2020-EPI_ISL_512070-2020-06-12
EPI_ISL_512071	29834	0	8909	5853	9589	5483	37.99691627	A	Lin-A-In-GJ-GBRC334b-2020-EPI_ISL_512071-2020-06-12
EPI_ISL_515954	29813	0	8902	5851	9576	5484	38.0203267	A	Lin-A-In-KA-nimh-13855-2020-EPI_ISL_515954-2020-05-09
EPI_ISL_482641	29863	3	8912	5858	9598	5482	37.97341191	B	Lin-B-In-DL-NCDC7554-CSIR-IGIB-2020-EPI_ISL_482641-2020-05-27
EPI_ISL_482661	29860	6	8914	5855	9588	5480	37.96048225	B	Lin-B-In-DL-NCDC7715-CSIR-IGIB-2020-EPI_ISL_482661-2020-05-28
EPI_ISL_483843	29836	0	8910	5855	9593	5478	37.98431425	B	Lin-B-In-GJ-GBRC255b-2020-EPI_ISL_483843-2020-06-12
EPI_ISL_483861	29837	0	8909	5858	9587	5483	38.00985354	B	Lin-B-In-GJ-GBRC266-2020-EPI_ISL_483861-2020-06-13
EPI_ISL_483877	29830	0	8908	5855	9585	5482	38.00536373	B	Lin-B-In-GJ-GBRC276-2020-EPI_ISL_483877-2020-06-13
EPI_ISL_486394	29836	1	8910	5856	9590	5479	37.99101756	B	Lin-B-In-KA-nimh-3720-2020-EPI_ISL_486394-2020-04-23
EPI_ISL_486399	29863	2	8917	5858	9598	5488	37.99350367	B	Lin-B-In-KA-nimh-7817-2020-EPI_ISL_486399-2020-04-29
EPI_ISL_486408	29839	0	8912	5856	9592	5479	37.98719796	B	Lin-B-In-KA-nimh-15819-2020-EPI_ISL_486408-2020-05-10
EPI_ISL_486853	29840	10	8911	5853	9592	5474	37.95911528	B	Lin-B-In-UN-UN2-2020-EPI_ISL_486853-2020-06-17
EPI_ISL_495035	29829	0	8905	5858	9584	5482	38.01669516	B	Lin-B-In-GJ-GBRC295-2020-EPI_ISL_495035-2020-06-15
EPI_ISL_495060	29825	0	8905	5854	9589	5477	37.99161777	B	Lin-B-In-GJ-GBRC308-2020-EPI_ISL_495060-2020-06-15
EPI_ISL_495174	29819	0	8903	5852	9591	5473	37.97914082	B	Lin-B-In-TG-CCMB-L1959-2020-EPI_ISL_495174-2020-06-04
EPI_ISL_495192	29819	0	8902	5853	9590	5474	37.98584795	B	Lin-B-In-TG-CCMB-M2-2020-EPI_ISL_495192-2020-06-04
EPI_ISL_495232	29819	0	8904	5850	9588	5477	37.98584795	B	Lin-B-In-TG-CCMB-M722-2020-EPI_ISL_495232-2020-06-11
EPI_ISL_495242	29819	0	8902	5852	9587	5478	37.99590865	B	Lin-B-In-TG-CCMB-M79-2020-EPI_ISL_495242-2020-06-05
EPI_ISL_495246	29819	0	8902	5852	9587	5478	37.99590865	B	Lin-B-In-TG-CCMB-M81-2020-EPI_ISL_495246-2020-06-05
EPI_ISL_495256	29819	0	8903	5850	9591	5475	37.97914082	B	Lin-B-In-TG-CCMB-M844-2020-EPI_ISL_495256-2020-06-12
EPI_ISL_495262	29819	0	8903	5852	9589	5475	37.98584795	B	Lin-B-In-TG-CCMB-M900-2020-EPI_ISL_495262-2020-06-13
EPI_ISL_495265	29819	0	8903	5852	9589	5475	37.98584795	B	Lin-B-In-TG-CCMB-M931-2020-EPI_ISL_495265-2020-06-13
EPI_ISL_495276	29835	0	8908	5855	9593	5479	37.98893917	B	Lin-B-In-TG-CCMB-OM10-2020-EPI_ISL_495276-2020-05-15
EPI_ISL_495281	29835	0	8910	5855	9588	5482	37.99899447	B	Lin-B-In-TG-CCMB-OM15-2020-EPI_ISL_495281-2020-05-17
EPI_ISL_508238	29898	0	8953	5861	9598	5486	37.9523714	B	Lin-B-In-MH-GA43-2020-EPI_ISL_508238-2020-06-20
EPI_ISL_508245	29903	0	8959	5857	9599	5488	37.93933719	B	Lin-B-In-MH-GA50-2020-EPI_ISL_508245-2020-06-20
EPI_ISL_508301	29903	0	8953	5862	9601	5487	37.95271377	B	Lin-B-In-KA-IB23-2020-EPI_ISL_508301-2020-06-21
EPI_ISL_508306	29881	0	8949	5858	9592	5482	37.95053713	B	Lin-B-In-KA-IB28-2020-EPI_ISL_508306-2020-06-20
EPI_ISL_508325	29876	0	8946	5859	9588	5483	37.96358281	B	Lin-B-In-KA-IB47-2020-EPI_ISL_508325-2020-06-03
EPI_ISL_508423	29901	0	8954	5861	9597	5489	37.9585967	B	Lin-B-In-MH-MW11-2020-EPI_ISL_508423-2020-04-29
EPI_ISL_508427	29879	0	8948	5855	9592	5484	37.94973058	B	Lin-B-In-MH-MW20-2020-EPI_ISL_508427-2020-05-15
EPI_ISL_508447	29877	8	8941	5859	9587	5482	37.95896509	B	Lin-B-In-WB-S39-2020-EPI_ISL_508447-2020-04-28
EPI_ISL_512064	29813	0	8901	5852	9584	5476	37.99684701	B	Lin-B-In-GJ-GBRC329-2020-EPI_ISL_512064-2020-06-25
EPI_ISL_512068	29790	0	8895	5850	9575	5470	37.99932863	B	Lin-B-In-GJ-GBRC332b-2020-EPI_ISL_512068-2020-07-27
EPI_ISL_515968	29798	0	8895	5852	9581	5470	37.99583865	B	Lin-B-In-KA-nimh-19510-2020-EPI_ISL_515968-2020-05-16
EPI_ISL_524713	29800	0	8895	5855	9577	5473	38.01342282	B	Lin-B-In-GJ-GBRC-362a-2020-EPI_ISL_524713-2020-06-18
EPI_ISL_524718	29800	0	8895	5853	9585	5467	37.98657718	B	Lin-B-In-GJ-GBRC-365-2020-EPI_ISL_524718-2020-06-18
EPI_ISL_524736	29762	0	8884	5850	9557	5471	38.03843828	B	Lin-B-In-GJ-GBRC-377b-2020-EPI_ISL_524736-2020-07-11
EPI_ISL_524741	29800	0	8897	5852	9582	5469	37.98993289	B	Lin-B-In-GJ-GBRC-381-2020-EPI_ISL_524741-2020-06-18
EPI_ISL_524763	29800	0	8895	5853	9579	5473	38.00671141	B	Lin-B-In-GJ-GBRC-394b-2020-EPI_ISL_524763-2020-07-06

EPI_ISL_528853	29818	0	8902	5850	9592	5474	37.97706084	B	Lin-B-In-TG-CCMB-X16-2020-EPI_ISL_528853-2020-07-14
EPI_ISL_528856	29818	0	8900	5855	9588	5475	37.99718291	B	Lin-B-In-TG-CCMB-X44-2020-EPI_ISL_528856-2020-07-14
EPI_ISL_528863	29818	0	8900	5849	9594	5475	37.97706084	B	Lin-B-In-TG-CCMB-X67-2020-EPI_ISL_528863-2020-07-14
EPI_ISL_539486	29809	0	8909	5853	9580	5467	37.97510819	B	Lin-B-In-PB-IMT-L176-2020-EPI_ISL_539486-2020-04-30
EPI_ISL_539618	29818	0	8900	5852	9590	5476	37.99047555	B	Lin-B-In-TG-CCMB-AB583-2020-EPI_ISL_539618-2020-08-16
EPI_ISL_539623	29818	8	8896	5852	9587	5475	37.98712187	B	Lin-B-In-TG-CCMB-R178-2020-EPI_ISL_539623-2020-08-09
EPI_ISL_539629	29818	0	8901	5852	9592	5473	37.98041451	B	Lin-B-In-TG-CCMB-AB520-2020-EPI_ISL_539629-2020-08-14
EPI_ISL_539636	29818	0	8903	5852	9592	5471	37.97370716	B	Lin-B-In-TG-CCMB-AA972-2020-EPI_ISL_539636-2020-08-13
EPI_ISL_539640	29818	0	8902	5852	9587	5477	37.99382923	B	Lin-B-In-TG-CCMB-AB223-2020-EPI_ISL_539640-2020-08-14
EPI_ISL_539643	29818	0	8902	5851	9590	5475	37.98376819	B	Lin-B-In-TG-CCMB-R305-2020-EPI_ISL_539643-2020-08-09
EPI_ISL_539650	29818	0	8901	5851	9591	5475	37.98376819	B	Lin-B-In-TG-CCMB-AA589-2020-EPI_ISL_539650-2020-08-12
EPI_ISL_539655	29818	0	8903	5849	9590	5476	37.98041451	B	Lin-B-In-TG-CCMB-AA669-2020-EPI_ISL_539655-2020-08-12
EPI_ISL_539658	29818	0	8900	5853	9592	5473	37.98376819	B	Lin-B-In-TG-CCMB-AC284-2020-EPI_ISL_539658-2020-08-19
EPI_ISL_539683	29818	0	8904	5851	9593	5470	37.9669998	B	Lin-B-In-TG-CCMB-AA105-2020-EPI_ISL_539683-2020-08-10
EPI_ISL_539688	29818	0	8903	5851	9591	5473	37.97706084	B	Lin-B-In-TG-CCMB-AA371-2020-EPI_ISL_539688-2020-08-11
EPI_ISL_539724	29818	5	8900	5852	9590	5471	37.97370716	B	Lin-B-In-TG-CCMB-R338-2020-EPI_ISL_539724-2020-08-09
EPI_ISL_539740	29818	0	8900	5851	9597	5470	37.9669998	B	Lin-B-In-TG-CCMB-AB227-2020-EPI_ISL_539740-2020-08-14
EPI_ISL_539745	29818	6	8896	5849	9592	5475	37.97706084	B	Lin-B-In-TG-CCMB-R286-2020-EPI_ISL_539745-2020-08-09
EPI_ISL_539747	29818	5	8898	5850	9592	5473	37.97370716	B	Lin-B-In-TG-CCMB-AA812-2020-EPI_ISL_539747-2020-08-13
EPI_ISL_539751	29821	0	8903	5850	9590	5478	37.9866537	B	Lin-B-In-TG-CCMB-AA241-2020-EPI_ISL_539751-2020-08-11
EPI_ISL_539775	29821	5	8898	5852	9591	5475	37.98330036	B	Lin-B-In-TG-CCMB-R325-2020-EPI_ISL_539775-2020-08-09
CoV_Riyadh_2016	29994	0	7857	6276	9793	6068	41.15489765	MERS	OL622035.1_MERS-CoV_Riyadh_2016

Supplementary Table 2. Dataset 2 has 69 SARS-Cov-2 random sequences of 8 strains obtained from GISAID (Name annotated with GISAID metadata information).

Name	Length	Name	Length
MU_VOI_B.1.621_GH_Illumina_USA	29356	Delta_B.1.617.2_GK_Iontor_England6	29834
MU_VOI_B.1.621_GH_PAcBio_USA2	29565	Delta_B.1.617.2_GK_IonTor_England_2	29834
MU_VOI_B.1.621_GH_PAcBio_USA3	29086	Beta_B.1.351.3_GH_IonTor_England43	29834
MU_VOI_B.1.621_GH_Nanopore_Colombia2	29493	Beta_B.1.351.3_GH_IonTor_England344	29834
MU_VOI_B.1.621_GH_Illumina_Chile2	29331	Beta_B.1.351_GH_IonTor_England234	29834
MU_VOI_B.1.621_GH_Illumina_Chile3	29565	Beta_B.1.351_GH_Illumina_Kenya4567	29898
MU_VOI_B.1.621_GH_Illumina_Chile4	29143	Beta_B.1.351_GH_Nanopore_Botswana43	29836
MU_VOI_B.1.621.2_GH_Illumina_Chile	29330	Lambda_C.37_GR_Illumina_Peru54	29863
MU_VOI_B.1.621_GH_PacBio_USA6	29565	Lambda_C.37_GR_Illumina_Peru_2	29901
MU_VOI_B.1.621_GH_Illumina_Chile6	29332	Lambda_C.37_GR_Illumina_USA87	29853
490R_B.1.640.1_GH_Nanopore_Rwanda	29655	Lambda_C.37_GR_nanopore_Ecuador23	29782
490R_B.1.640.1_GH_Nanopore_Canada	29665	Lambda_C.37_O_Illumina_Chile345	29306
490R_B.1.640.1_GH_Nanopore_Belgium	29713	Gamma_P.1.14_GR_Illumina_Turkey345	29813
490R_B.1.640.1_GH_Illumina_France3	29816	Gamma_P.1_GR_Illumina_France765	29903
490R_B.1.640.1_GH_Illumina_France6	29816	Gamma_P.1_GR_IonTor_Spain909	29903

490R_B.1.640.1_GH_Illumina_France7	29815	Gamma_P.1.1_GR_Illumina_Italy789	29903
490R_B.1.640.1_GH_Illumina_France8	29816	Gamma_P.1.10_GR_Illumina_Costa_Rica345	29746
490R_B.1.640.1_GH_Illumina_France10	29815	Gamma_P.1_GR_Illumina_Costa_Rica5	29778
490R_B.1.640.1_Nanopore_Nigeria	29631	Gamma_P.1.1_GR_Illumina_Italy67	29878
OL622035.1_MERS-CoV_Riyadh_2016	29994	Omnicron_BA.1.18_GRA_Nanopore_Botswana3647	29714
Alpha_B.1.1.7_GR_Illumina_Ind145	29892	Omnicron_BA.1.18_GRA_Nanopore_Botswana2	29670
alpha_B.1.1.7_GR_ION_torront_Spain1	29903	Omnicron_BA.1.17.2_GRA_Nanopore_India68	29746
alpha_B.1.1.7_GR_Illumina_Ind2462	29815	Omnicron_BA.1.1.11_GRA_Illumina_Switzerland2	29833
alpha_B.1.1.7_GR_ION_torront_Spain233	29903	Omnicron_BA.1.17_GRA_CLC_USA037	29502
alpha_B.1.1.7_GR_Illumina_Spain3	29903	Omicron_USA_EPI_ISL_10430488	29747
alpha_B.1.1.7_GR_Illumina_Egypt1	29903	Omicron_USA_EPI_ISL_10430497	29747
alpha_B.1.1.7_GR_Illumina_Spain5	29903	Omicron_USA_EPI_ISL_10430530	29747
alpha_B.1.1.7_G_nanopore_Belgium_travel1	29772	Omicron_USA_EPI_ISL_10430542	29747
Delta_B.1.617.2_GK_Illumina_Ind6	29817	Omicron_India_KA_EPI_ISL_10746622	29903
Delta_B.1.617.2_GK_Illumina_Ind_2	29817	Omicron_India_EPI_ISL_10746635	29903
Delta_AY.122_GK_Illumina_Germany3	29903	Omicron_India_EPI_ISL_10746648	29903
Delta_AY.122_GK_Illumina_Germany_2	29903	Omicron_India_EPI_ISL_10746652	29903
Delta_AY.122_GK_Iontor_Spain88	29903	Omicron_India_EPI_ISL_10746663	29903
Delta_AY.122_GK_Iontor_Spain_2	29903	Omicron_India_EPI_ISL_10746666	29903
		Omicron_Germany_EPI_ISL_9720320	29798

Supplementary Table 3. Dataset 3 made up of 50 random sequences of Omicron sublineages, BJ.1 and BA.2.75 and recently discovered XBB, a recombinant lineage between two Omicron sublineages, BJ.1 and BA.2.75.

	length	n_base	a_base	g_base	t_base	c_base	GC_content	SubLineage/ recombinant
XBB.1_EPI_ISL_15209055|2022-09-27	29831	14	8927	5838	9591	5460	37.87335322	XBB.1
XBB.1_EPI_ISL_15209059|2022-09-27	29831	24	8921	5837	9592	5456	37.85659214	XBB.1
XBB.1_EPI_ISL_15209060|2022-09-26	29831	24	8922	5837	9587	5459	37.86664879	XBB.1
XBB.1_EPI_ISL_15209061|2022-09-27	29831	26	8920	5836	9588	5460	37.86664879	XBB.1
XBB.1_EPI_ISL_15209062|2022-09-27	29831	24	8922	5835	9590	5457	37.85323992	XBB.1
XBB.1_EPI_ISL_15209063|2022-09-27	29828	23	8921	5835	9589	5458	37.86039962	XBB.1
XBB.1_EPI_ISL_15209064|2022-09-27	29827	25	8921	5834	9588	5458	37.85831629	XBB.1
XBB.1_EPI_ISL_15209066|2022-09-28	29832	30	8921	5836	9586	5456	37.85197104	XBB.1
XBB.1_EPI_ISL_15209073|2022-09-26	29823	30	8916	5834	9583	5459	37.86674714	XBB.1
XBB.1_EPI_ISL_15209076|2022-09-27	29818	25	8918	5832	9581	5457	37.85968207	XBB.1
XBB.1_EPI_ISL_15209086|2022-09-26	29803	22	8913	5832	9584	5450	37.85524947	XBB.1
XBB.1_EPI_ISL_15209101|2022-09-27	29773	12	8893	5830	9582	5455	37.90346959	XBB.1
XBB.1_EPI_ISL_15209105|2022-09-27	29788	30	8907	5828	9575	5447	37.85081241	XBB.1
ba.2.75_EPI_ISL_13905312|2022-07-01	29724	0	8882	5831	9580	5431	37.88857489	ba.2.75
ba.2.75_EPI_ISL_14285381|2022-07-28	29381	0	8790	5753	9478	5360	37.82376366	ba.2.75
ba.2.75_EPI_ISL_14285457|2022-07-24	29381	0	8788	5751	9481	5361	37.8203601	ba.2.75
ba.2.75_EPI_ISL_14285512|2022-07-23	29372	0	8786	5752	9475	5359	37.82854419	ba.2.75

ba.2.75_EPI_ISL_14285531|2022-07-25	29381	0	8788	5753	9480	5360	37.82376366	ba.2.75
ba.2.75_EPI_ISL_14359504|2022-08-02	29749	0	8887	5833	9591	5438	37.8869878	ba.2.75
ba.2.75_EPI_ISL_14434715|2022-08-01	29381	0	8786	5755	9478	5362	37.8373779	ba.2.75
ba.2.75_EPI_ISL_14536448|2022-08-07	29381	0	8788	5753	9477	5363	37.83397434	ba.2.75
ba.2.75_EPI_ISL_14536600|2022-08-09	29381	0	8788	5754	9482	5357	37.81695654	ba.2.75
ba.2.75_EPI_ISL_14548110|2022-08-01	29810	37	8918	5833	9584	5438	37.80945991	ba.2.75
ba.2.75_EPI_ISL_14548509|2022-08-06	29847	33	8932	5838	9596	5448	37.81284551	ba.2.75
ba.2.75_EPI_ISL_14548958|2022-08-08	29848	35	8933	5838	9597	5445	37.80152774	ba.2.75
ba.2.75_EPI_ISL_14632919|2022-07-26	29652	1	8858	5814	9559	5420	37.88614596	ba.2.75
ba.2.75_EPI_ISL_14633477|2022-08-13	29652	19	8854	5809	9552	5418	37.86253878	ba.2.75
ba.2.75_EPI_ISL_14633808|2022-07-19	29652	1	8857	5813	9559	5422	37.88951841	ba.2.75
ba.2.75_EPI_ISL_14633933|2022-07-24	29652	1	8859	5813	9556	5423	37.89289087	ba.2.75
ba.2.75_EPI_ISL_14634153|2022-08-01	29652	1	8858	5813	9558	5422	37.88951841	ba.2.75
ba.2.75_EPI_ISL_14634490|2022-08-12	29652	2	8858	5811	9558	5423	37.88614596	ba.2.75
ba.2.75_EPI_ISL_14634492|2022-08-12	29652	1	8858	5811	9560	5422	37.88277351	ba.2.75
ba.2.75_EPI_ISL_14634634|2022-07-10	29652	1	8858	5814	9556	5423	37.89626332	ba.2.75
ba.2.75_EPI_ISL_14634841|2022-07-16	29652	1	8858	5812	9559	5422	37.88614596	ba.2.75
ba.2.75_EPI_ISL_14635906|2022-07-16	29652	1	8858	5814	9557	5422	37.89289087	ba.2.75
ba.2.75_EPI_ISL_14636227|2022-07-30	29652	1	8859	5813	9559	5420	37.88277351	ba.2.75
ba.2.75_EPI_ISL_14636498|2022-08-07	29652	1	8857	5814	9559	5421	37.88951841	ba.2.75
BJ.1_EPI_ISL_14303196|2022-07-22	29750	0	8893	5826	9580	5451	37.90588235	BJ.1
BJ.1_EPI_ISL_14459008|2022-08-08	29693	0	8876	5816	9562	5436	37.89445324	BJ.1
BJ.1_EPI_ISL_14733814|2022-07-30	29762	31	8886	5823	9576	5446	37.86371884	BJ.1
BJ.1_EPI_ISL_14750054|2022-08-24	29754	0	8893	5837	9576	5448	37.92767359	BJ.1
BJ.1_EPI_ISL_14891585|2022-08-05	29726	1	8885	5826	9572	5442	37.90621005	BJ.1
BJ.1_EPI_ISL_14917666|2022-09-07	29800	27	8911	5829	9583	5447	37.83892617	BJ.1
BJ.1_EPI_ISL_14917739|2022-09-07	29736	32	8894	5818	9559	5432	37.83292978	BJ.1
BJ.1_EPI_ISL_14933770|2022-09-02	29721	0	8885	5823	9572	5439	37.89239931	BJ.1
BJ.1_EPI_ISL_14986875|2022-08-27	29739	0	8888	5827	9581	5443	37.89636504	BJ.1
BJ.1_EPI_ISL_15050448|2022-08-11	29649	0	8860	5808	9550	5431	37.9068434	BJ.1
BJ.1_EPI_ISL_15157662|2022-09-20	29757	0	8891	5829	9585	5452	37.91040764	BJ.1
BJ.1_EPI_ISL_15398315|2022-09-19	29724	0	8885	5822	9577	5440	37.88857489	BJ.1
OL622035.1_MERS-CoV_Riyadh_2016	29994	0	7857	6276	9793	6068	41.15489765	OL622035.1

Supplementary Material for CGR Method

Details of Chaos Game Representation (CGR) Method

The Chaos Game Representation (CGR) is a powerful method for visually representing genomic sequences in two dimensions. It converts linear nucleotide sequences into dot plots, offering a compact and informative way to compare genomes. H.J. Jeffery first introduced this algorithm for visualizing genomic sequences in 1990 [1] (Jeffery, 1990). Originally used solely for visualization, CGR later found applications in studying phylogenetic relationships among diverse species [2] (Almeida, 2001). Additionally, CGR has been employed to explore intra-species variability, such as classifying HIV-1 subtypes using the CGR method with k=6 [3] (Pandit, Sinha; 2010).

Constructing a CGR Plot (Mapping x, y coordinates of nucleotides): To create a CGR plot, a square box of specific dimensions is chosen. In our program, we defined it as the square root of the 4^K for the implementation purpose. However, dimensions of the square (frequency matrix dimension) could be any fixed value. For instance, if we consider K=2, a square of 4*4 dimensions is plotted, with corners labelled as A (coordinates 0, 0), T (4, 0), G (4, 4), and C (0, 4), representing the four nucleotides. The midpoint of the square (i.e., 2, 2) serves as the starting point, and nucleotides from the genomic sequence are read sequentially. For example, if the input genomic sequence is "ATTCG," the first dot is plotted at x = (2+0)/2=1 and y = (2+0)/2=1, i.e., the midpoint between the A corner and the starting point. The next dot is then plotted for the nucleotide "T" by taking the midpoint between the previous point (1, 1) and the T corner (4, 0), resulting in x= (1+4)/2=2.5 and y= (1+0)/2=0.5. This process continues for each nucleotide, plotting dots sequentially until the entire sequence is represented, resulting in a CGR plot (as shown in the figure here). The arrows show the direction of reading the DNA sequence (from left to right).

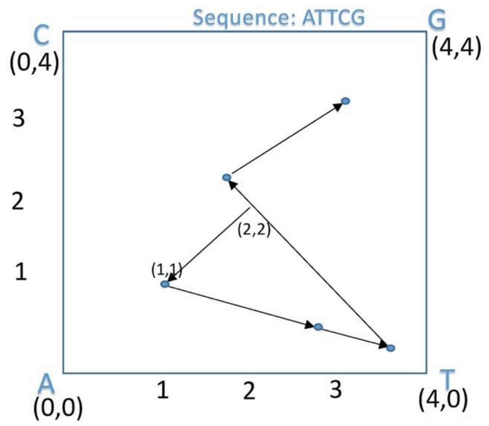

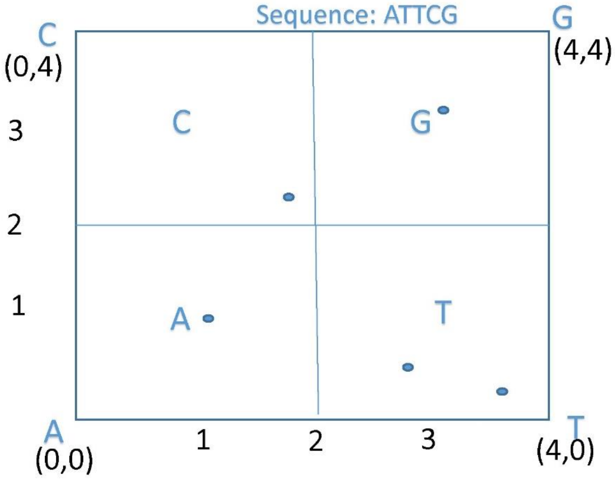

The CGR plot can be interpreted in a unique way. For example, if we set k=1, we count the number of dots in each of the four sub-squares and store the results in a 2x2 matrix. See figure with four subdivisions. This matrix is called the **frequency matrix**. For k=2 (di-mer), the matrix becomes 4x4. We can use the CGR plot to derive di-nucleotides (dimers), tri-nucleotides (trimers), and even higher nucleotide sequences (k-mers) for any genome sequence. To calculate the frequency of each k-mer, the CGR square is divided into a 4^k grid. The number of points plotted in a specific grid reflects the frequency of that particular k-mer in the genome sequence.

Another fascinating property of the CGR plot is its **fractal nature**. The iterative process of plotting points on the CGR plot creates intricate and self-similar patterns at different scales. This means that each square box in the plot contains a smaller version of the entire plot, exhibiting similarity to the overall pattern. This characteristic of self-replication is typical of fractals, complex geometric structures that reveal repeating patterns at various levels of magnification.

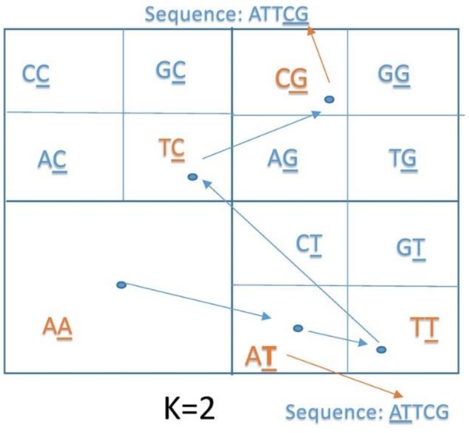

As we can see in the figure here, if we divide square based on k=2, each dot in the sub-square, retains the patterns of previous nucleotide in it. As the genomic sequence is traversed and each nucleotide is plotted as a point on the CGR, the pattern unfolds in a fractal-like manner, with smaller elements mirroring the larger ones. This fractal nature allows researchers to explore genomic sequences at multiple resolutions, facilitating the examination of both local and global patterns in the DNA.

Algorithm implementation: The main function of the algorithm, known as the "cgat function," operates as follows:

- **Read Nucleotides:** The algorithm reads each nucleotide from the given DNA sequence one by one.
- **Determine Sub-Square:** For each nucleotide, the algorithm automatically determines the corresponding sub-square within the CGR plot where it belongs.
- **Counting Hits:** The algorithm keeps track of the hits by saving them as counts. Whenever a nucleotide is plotted in a sub-square, the count for that sub-square is increased by 1.
- **Accumulative Count:** As the algorithm progresses and encounters additional nucleotides that belong to the same sub-square, it incrementally increases the count for that particular sub-square.

The figure presented below illustrates the words along with their corresponding counts for k=3 (total of 64 words), presented in the form of a frequency matrix:

CCC	GCC	CGC	GGC	CCG	GCG	CGG	GGG
ACC	TCC	AGC	TGC	ACG	TCG	AGG	TGG
CAC	GAC	CTC	GTC	CAG	GAG	CTG	GTG
AAC	TAC	ATC	TTC	AAG	TAG	ATG	TTG
CCA	GCA	CGA	GGA	CCT	GCT	CGT	GGT
ACA	TCA	AGA	TGA	ACT	TCT	AGT	TGT
CAA	GAA	CTA	GTA	CAT	GAT	CTT	GTT
AAA	TAA	ATA	TTA	AAT	TAT	ATT	TTT

78	73	15	117	15	26	26	186
110	74	192	94	20	19	238	199
98	126	83	55	274	2610	126	95
146	125	122	66	283	204	187	117
144	207	28	255	98	112	11	91
249	138	321	119	116	95	174	113
224	274	130	157	142	159	82	82
402	204	231	157	273	142	176	137

Calculation of distances between various sequences: Once frequency matrices are generated for each input sequence at specified K value, distances between various sequences can be computed using simple *Euclidian* distance, Euclidean squared distance and the *Manhattan* distance distance methods. A distance matrix between sequences is useful for phylogenetic analysis. Distance Matrix between two genome sequences is computed by calculating the difference in the frequencies of k-mers from their CGR plots. Significant differences in the whole genome sequence length and presence of n/N nucleotide bases (genomic location where software not able to make a basecall are usually listed as "N" base) contributes to frequency bias. Since we are analyzing closely related genomes, to minimize such bias, the end bases are trimmed to obtain the same length of every sequence equal to the minimum length of the sequence in the input dataset.

The simple Euclidian distance matrix between two genome sequences is computed by calculating the difference in the frequencies of k-mers of their CGR plots. A simple way to calculate the distance matrix is to use the Euclidean distance given by –

$$d(\mathrm{N},\mathrm{M}) = \sqrt{\sum_{i=1}^{4^k}(n_i - m_i)^2}$$

Where, n_i is the count of ith word in genome N and m_i is the count of ith word in genome M. For Manhattan distance formula changes to following:

$$d(\mathrm{N},\mathrm{M}) = \sum_{i=1}^{4^k}|n_i - m_i|$$

Distance calculation task is performed by matrixDistance function. Which reads frequency matrix of two genomes side by side.

Saving Results in different output formats (Mega, Phylip, Newick, Nexus)

For tree visualization with third-party tools, results can be saved into Mega or Phylip format using save MegaDistance and savePhylipDistance respectively. Outtree, users can save outtree files created by the NJ method of Bioconductor package 'ape'. These files contain information about trees generated in standard NEWICK format or other. These files are compatible with standard bioinformatics tools like TreeView, MEGA, etc. to create, view, edit, and customize trees.

REFERENCES

[1] Jeffrey, H.J. Chaos game representation of gene structure. Nucleic Acids Res., 1990, 18(8), 2163-2170. http://dx.doi.org/10.1093/nar/18.8.2163 PMID: 2336393

[2] Almeida, J.S.; Carriço, J.A.; Maretzek, A.; Noble, P.A.; Fletcher, M. Analysis of genomic sequences by Chaos Game Representation. Bioinformatics, 2001, 17(5), 429-437. http://dx.doi.org/10.1093/bioinformatics/17.5.429 PMID: 11331237

[3] Pandit, A.; Sinha, S. Using genomic signatures for HIV-1 subtyping. BMC Bioinformatics, 2010, 11(S1)(Suppl. 1), S26. http://dx.doi.org/10.1186/1471-2105-11-S1-S26 PMID: 20122198
